# Revising the Intolerance of Uncertainty Model of Generalized Anxiety Disorder: Evidence from UK and Italian Undergraduate Samples

**DOI:** 10.3389/fpsyg.2016.01723

**Published:** 2016-11-01

**Authors:** Gioia Bottesi, Marta Ghisi, Eleonora Carraro, Nicola Barclay, Rebecca Payne, Mark H. Freeston

**Affiliations:** ^1^Department of General Psychology, University of Padua, PaduaItaly; ^2^Northumberland, Tyne and Wear NHS Foundation Trust, Newcastle upon TyneUK; ^3^School of Psychology, Newcastle University, Newcastle upon TyneUK

**Keywords:** intolerance of uncertainty, worry, somatic anxiety, mediation, moderation, analog samples

## Abstract

The Intolerance of Uncertainty Model (IUM) of Generalized Anxiety Disorder (GAD) attributes a key role to Intolerance of Uncertainty (IU), and additional roles to Positive Beliefs about Worry (PBW), Negative Problem Orientation (NPO), and Cognitive Avoidance (CA), in the development and maintenance of worry, the core feature of GAD. Despite the role of the IUM components in worry and GAD has been considerably demonstrated, to date no studies have explicitly assessed whether and how PBW, NPO, and CA might turn IU into worry and somatic anxiety. The current studies sought to re-examine the IUM by assessing the relationships between the model’s components on two different non-clinical samples made up of UK and Italian undergraduate students. One-hundred and seventy UK undergraduates and 488 Italian undergraduates completed measures assessing IU, worry, somatic anxiety, depression, and refined measures of PBW, NPO, and CA. In each sample, two mediation models were conducted in order to test whether PBW, NPO, and CA differentially mediate the path from IU to worry and the path from IU to somatic anxiety. Secondly, it was tested whether IU also moderates the mediations. Main findings showed that, in the UK sample, only NPO mediated the path from IU to worry; as far as concern the path to anxiety, none of the putative mediators was significant. Differently, in the Italian sample PBW and NPO were mediators in the path from IU to worry, whereas only CA played a mediational role in the path from IU to somatic anxiety. Lastly, IU was observed to moderate only the association between NPO and worry, and only in the Italian sample. Some important cross-cultural, conceptual, and methodological issues raised from main results are discussed.

## Introduction

Intolerance of Uncertainty (IU) can be defined as the “individual’s dispositional incapacity to endure the aversive response triggered by the perceived absence of salient, key, or sufficient information, and sustained by the associated perception of uncertainty” (Carleton, 2016, p. 31); individuals high in IU find situations that are uncertain threatening, upsetting, and undesirable, regardless of the actual probability of a negative event to occur (Dugas et al., 1998). IU as a construct was originally developed by the Laval team working on models and treatments of generalized anxiety disorder (GAD) in the early nineties (Freeston et al., 1994; Dugas et al., 1998). Among the systematically validated explanatory models of GAD (for a complete review, refer to Behar et al., 2009), the Intolerance of Uncertainty Model of GAD (IUM) proposed by Dugas et al. (1998) originally asserted that four factors contribute to the development and the maintenance of worry, the core feature of GAD (American Psychiatric Association [APA], 2013): IU, Positive Beliefs about Worry (PBW), Negative Problem Orientation (NPO), and Cognitive Avoidance (CA).

A robust body of research bolstered the evidence of IU, the model’s main feature, as a cognitive vulnerability factor for worry, as well as a maintaining factor for GAD (e.g., Ladouceur et al., 2000; Sexton et al., 2003; Koerner and Dugas, 2008). Negative beliefs about uncertainty usually interfere with the individual’s ability to effectively deal with these situations and promote the use of worry as a dysfunctional strategy to cope with or prevent feared outcomes (Dugas et al., 1998; Behar et al., 2009). PBW are distorted beliefs about the usefulness of worry; false contingencies usually act as both positive (e.g., worrying can sometimes produce effective solutions) and negative (the non-occurrence of a feared event) reinforcements maintaining the use of worry (Freeston et al., 1994; Dugas et al., 1998). Indeed, individuals endorsing PBW believe that worry is a positive personality feature (i.e., “being a worrier means being thoughtful”), and that worrying is an effective problem solving strategy, capable of preventing negative situations to occur, avoiding unpleasant emotions associated with negative events, and motivating to act in life (Freeston et al., 1994). NPO refers to a negative attitude toward problems and consists of a set of negative cognitive and emotional reactions that are activated when a problem situation occurs. It is associated with low levels of confidence about the ability to solve problems successfully, low personal control over the problem solving process, and pessimism about problem-solving outcomes (D’Zurilla and Nezu, 1999, 2006). When facing a problem situation, individuals with GAD usually do not focus on a problem solving strategy and do not consider themselves as effective problem solvers although they have an adequate knowledge of problem solving skills (Dugas et al., 1995, 1997; Robichaud and Dugas, 2005b; Koerner and Dugas, 2006). Lastly, CA is a cognitive process endorsed to avoid and/or suppress unwanted mental content, especially fearful mental images inducing peripheral physiological activation (Borkovec et al., 1993, 2004). CA includes a number of strategies (both automatic and controlled) such as substituting threatening thoughts with neutral or positive ones, transforming mental images into verbal-linguistic thoughts, etc., and it is particularly relevant to GAD (Dugas et al., 1998; Dugas and Koerner, 2005). Notably, since avoidance impairs the emotional processing of fear (Foa and Kozak, 1986), CA usually leads to the maintenance of high levels of worry and anxiety (Dugas et al., 1998; Borkovec et al., 2004). Importantly, the clinical efficacy of the IUM-based treatments for GAD relative to wait-list control conditions (Dugas and Ladouceur, 2000; Ladouceur et al., 2000; Dugas et al., 2003, 2010; Dugas and Robichaud, 2007) has been supported in multiple studies.

Despite the role of all components in worry and GAD has been considerably demonstrated (e.g., Freeston et al., 1994; Borkovec and Roemer, 1995; Dugas et al., 1997, 2005; Ladouceur et al., 1998, 1999; Robichaud et al., 2003; Buhr and Dugas, 2006; Koerner and Dugas, 2008), only a few studies were conducted to shed light on the mechanisms through which the IUM components act in determining worry and somatic anxiety levels, i.e., the core features of GAD. For example, Dugas et al. (2007) examined the associations between the IUM components and different indicators of severity (diagnostic severity, worry severity, and somatic symptom severity) in patients with a primary diagnosis of GAD. Results from correlations showed that IU and NPO were correlated with all the three indicators of severity, whereas CA was related with diagnostic and somatic symptom severity and PBW were only associated with diagnostic severity. Furthermore, when age, gender, and depressive symptoms were controlled, IU and NPO maintained their positive correlations with worry, and IU showed stronger correlations with worry severity rather than with somatic symptom severity. Lastly, the conduction of linear regressions highlighted that, partialling out the contribution of IU, NPO was no longer a significant predictor of diagnostic severity, worry severity, and somatic symptom severity, thus suggesting that the role of IU in determining worry levels is more relevant than the role played by the other variables (Dugas et al., 2007). Nonetheless, the correlational nature of this study does not allow understanding how the IUM components interact in producing worry and somatic anxiety. A subsequent attempt to examine a mediational path from IU to worry is represented by a study by Koerner and Dugas (2008), who tested the mediating role of appraisals of ambiguous situations in the relationship between IU and worry through a vignette task. University students high and low in IU were compared on their appraisals of ambiguous, negative, and positive situations; the former appraised all the situations they were presented as more distressing than the latter (and this was particularly true for ambiguous ones). Furthermore, the authors observed that appraisals (cognitive bias) partially mediated the path from IU to worry, and that worry partially mediated the path from IU to appraisals. Despite results provided support to the hypothesis of reciprocal relationships between worry and cognitive processes, the other IUM components were not taken into account in this study (Koerner and Dugas, 2008).

### The Current Studies

In the last decade a fast growing interest in the role of IU as a shared vulnerability factor for emotional disorders (e.g., Gentes and Ruscio, 2011; Carleton, 2012) has been established, and evidence supporting its involvement in several psychopathologies has been reported (e.g., Holaway et al., 2006; Boelen and Reijntjes, 2009; van der Heiden et al., 2010; McEvoy and Mahoney, 2012; Carleton et al., 2013; Paulus et al., 2015; Banducci et al., 2016; Jensen et al., 2016; Oglesby et al., 2016). Nonetheless, since the development of the IUM of GAD in 1998, the interest on IU as a factor potentially specific to the development and maintenance of worry and GAD has not diminished and the vulnerability role played by IU in the development and maintenance of worry and GAD has been repeatedly demonstrated (please refer to Koerner and Dugas, 2008). For example, whilst all IUM components have been shown to contribute to GAD as previously described, IU has been identified as the strongest predictor of GAD in adults (e.g., Dugas et al., 1997, 2007) as well as in adolescents (e.g., Laugesen et al., 2003); the experimental manipulation of IU has been shown to induce changes in worry, with higher IU leading to more worry (e.g., Ladouceur et al., 2000; Koerner and Dugas, 2008); psychological interventions for GAD targeting increasing tolerance of uncertainty have been demonstrated to reduce worry (e.g., Dugas and Ladouceur, 2000; Ladouceur et al., 2000; Dugas et al., 2003).

Despite such a shift in the conceptualization of IU (from disorder-specific variable to transdiagnostic factor), we believe that gaining a better understanding of the mechanisms through which IU operates within specific conditions, leading to specific kinds of phenomenology, might help clarifying how processes may be specifically enacted in a way close to the expression of symptoms. Therefore, the main aim of the present study was to re-examine the original IUM model and, taking into account the previously mentioned evidence supporting the dominance and precedence of IU on worry/GAD (please refer to Koerner and Dugas, 2008), we sought to further investigate whether and how the other IUM components, i.e., PBW, NPO, and CA, turn IU into worry and somatic anxiety. We decided to pursue this aim by testing a refined IUM model on two different non-clinical samples made up of UK and Italian undergraduate students in light of the following reasons.

First, research on analog samples could be appropriate when advancing theories and models aimed at understanding clinical phenomena, also because the phenotypic heterogeneity characterizing clinical populations, as well as the existence of comorbidity or treatment confounds, could represent a possible obstacle to the investigation of the etiology of psychological disorders (e.g., Abramowitz et al., 2014); furthermore, several authors demonstrated the dimensional latent structure of worry and anxiety symptoms in the population (e.g., Ruscio et al., 2001; Olatunji et al., 2010). To note, a significant part of the model’s development and subsequent testing has been conducted on undergraduate samples (e.g., Freeston et al., 1994; Buhr and Dugas, 2002; Carleton et al., 2007; Koerner and Dugas, 2008; Helsen et al., 2013).

Second, we sought to preliminary investigate whether such a refined IUM model could be applicable across cultures. Notably, evaluating the existence of cross-cultural differences when dealing with depression and anxiety is generally encouraged (e.g., Malgady, 1996; Norton, 2005), since environmental factors may vary across different cultures and cultural values might play a crucial role in defining the way individuals interpret and express their emotions and symptoms (Kirmayer, 2001). Despite UK and Italy are relatively close European countries, thus potentially sharing a number of similarities, they are also characterized by some cultural differences which might reasonably affect the way people of different cultures understand and express uncertainty, IU, worry and anxiety. For example, Italian people have been reported to be more prone to show their emotions, even when negative, than British (Lecce and Hughes, 2010). Even more interestingly, studies in the field of economics and business sciences have classified Italy among the “strong uncertainty avoidance cultures,” whereas the UK has been included within the “weak uncertainty avoidance” ones (De Mooij, 2000, 2003; Stremersch and Tellis, 2004; Wennekers et al., 2007). In this field, “uncertainty avoidance” refers to “the extent to which the members of a culture feel threatened by uncertain or unknown situations […] “In uncertainty avoidant cultures, risk taking is limited to known risks (of which the probability is known), while in cultures low in uncertainty avoidance, risk taking includes unknown risks (of which the probability is not known)” (Stremersch and Tellis, 2004, p. 426). According to this, Italian people are likely to be more conservative and more resistant to change than UK individuals.

In light of the above-mentioned issues, we re-examined the Laval model for GAD by conducting two studies testing two different mediation models in two different undergraduate samples (UK and Italian). In particular, we aimed at testing whether PBW, NPO, and CA differentially mediate the path from IU to worry and the path from IU to somatic anxiety. We expected that:

(1) PBW, in light of their content specificity to worry (Freeston et al., 1994), would mediate the relationship between IU and worry and not between IU and somatic anxiety;
(2) NPO would mediate the paths from IU to both worry and somatic anxiety. Indeed, findings by Dugas et al. (2007) suggested that IU and NPO could be somewhat considered equally strong predictors of worry and somatic anxiety severity, and NPO has been suggested to be relevant across anxiety symptoms (Fergus and Wu, 2011). The same was expected also for CA, given previous evidence (Dugas et al., 2007) and that it is usually endorsed to decrease somatic activity (Borkovec et al., 2004).
(3) In light of evidence supporting that higher IU leads to higher levels of worry (e.g., Ladouceur et al., 2000; Koerner and Dugas, 2008), we decided to assess whether IU also moderates the mediations, i.e., whether the contribution of PBW, NPO and CA not only stems from IU, but depends on the level of IU.

Lastly, since no previous studies comparing the IUM components across UK and Italian undergraduate samples were conducted, we decided to exploratory investigate the presence of any similarities and differences between groups without advancing specific hypotheses. However, keeping in mind that the British and the Italian cultures are claimed to differ in terms of “uncertainty avoidance” (De Mooij, 2000, 2003; Stremersch and Tellis, 2004; Wennekers et al., 2007), we speculated that potential differences in the tested models could reasonably emerge.

## Study 1

### Materials and Methods

#### Participants and Procedure

One hundred and seventy (86.5% females) UK undergraduate students entered Study 1. Participants were all students attending their 1st year aged between 18 and 30 years (*M* = 19.14; *SD* = 1.50), 94.4% were Caucasian, and they were recruited at the School of Psychology of the Newcastle University (United Kingdom) during University courses; no incentives were offered for participation. All participants were informed of the study’s aims and gave their written informed consent before completing the battery of questionnaires, which were administered in rotated order to control for order effect. The research was approved by the Ethics Committee of the Faculty of Medical Sciences (Newcastle University).

#### Measures

The *Intolerance of Uncertainty Scale-12* (IUS-12; Carleton et al., 2007) is a 12-item self-report measure designed to assess the individual’s tendency to find uncertainty upsetting and distressing. Items on the IUS-12 were derived from the original IUS (Freeston et al., 1994; Buhr and Dugas, 2002) and are highly correlated with the original scale. Respondents are asked to evaluate the extent to which each statement applies to them on a 5-point Likert scale ranging from 1 = “not at all characteristic of me” to 5 = “entirely characteristic of me.” The IUS-12 has demonstrated excellent internal consistency, convergent, and discriminant validity (Carleton et al., 2007; Khawaja and Yu, 2010; McEvoy and Mahoney, 2011). In line with the aims of the present study, the Total score was used; internal consistency in the current sample was excellent (α = 0.93).

The *Why Worry-III* (WW-III; Riley, 2010) is a 37-item revised version of the Why Worry questionnaire (Freeston et al., 1994) and the Why Worry-II (Gosselin et al., 2003) designed to assess PBW. The WW-III requires respondents to think back to a time when they have worried and rate the extent to which they agree to each item on a 5-point Likert scale ranging from 1 = “disagree a lot” to 5 = “agree a lot”. Seven different subscales, each corresponding to seven PBW, were identified (Riley, 2010). Given the goals of the present study, only the WW-III total score was retained for the analyses. Similar to previous versions, also the WW-III showed good psychometric properties (Riley, 2010); in the current sample, Cronbach alpha for the total score was excellent (α = 0.94).

The *Negative Problem Orientation Questionnaire* (NPOQ; Robichaud and Dugas, 2005a,b) is a 12-item questionnaire assessing an individual’s approach to problems, including beliefs that problems are threatening, low self-confidence about abilities to solve problems, and pessimism about problem resolution (Robichaud and Dugas, 2005a). Respondents are required to rate the extent to which each statement corresponds to the way they think about problems on a 5-point Likert scale ranging from 1 = “not at all true of me” to 5 = “extremely true of me.” The NPOQ has shown adequate internal consistency, test–retest reliability, convergent, and discriminant validity (Robichaud and Dugas, 2005a,b). The internal consistency observed in the present sample was excellent (α = 0.96).

The *Revised Cognitive Avoidance Questionnaire* (R-CAQ; Heary, 2011) The R-CAQ is a 35-item revised version of the Cognitive Avoidance Questionnaire (Gosselin et al., 2002) and it is a self-report measure designed to assess an individual’s use of seven CA strategies. Participants have to rate the extent to which each item is a typical mental action they perform in response to certain thoughts on a 5-point Likert scale ranging from 1 = “disagree a lot” to 5 = “agree a lot.” As for the WW-III, given the aim of the present study, only the R-CAQ total score was considered in the analyses. As for the original version, the R-CAQ has demonstrated excellent psychometric properties (Heary, 2011) and, in the current sample, internal consistency was excellent (α = 0.94).

The *Penn State Worry Questionnaire* (PSWQ; Meyer et al., 1990) is a 16-item inventory designed to assess the tendency to worry excessively and uncontrollably. It measures trait worry by asking individuals to rate the extent to which each item is typical of them on a 5-point Likert scale ranging from 1 = “not at all typical of me” to 5 = “very typical of me.” Internal consistency and test–retest reliability of the PSWQ were good in non-clinical and clinical samples (Meyer et al., 1990). Furthermore, convergent and divergent validity were good (Molina and Borkovec, 1994). In the current sample, Cronbach alpha emerged to be adequate (α = 0.78).

The *Depression Anxiety Stress Scales -21* (DASS-21; Lovibond and Lovibond, 1995) is a 21-item questionnaire organized into three scales: Depression, referring to lack of incentive, low self-esteem, and dysphoria; anxiety, assessing somatic and subjective symptoms of anxiety, as well as acute responses of fear; and stress, measuring irritability, impatience, tension, and persistent arousal. Respondents have to indicate the extent to which each statement applies to them over the previous week on a 4-point Likert scale ranging from 0 = “did not apply to me at all” to 3 = “applied to me very much, or most of the time.” Good internal consistency, convergent and discriminant validity on a large UK non-clinical sample were documented (Henry and Crawford, 2005). Given the purposes of the present study, only the Anxiety and Depression scales were considered in the analyses; observed Cronbach alphas values in the current sample were good (α = 0.85 and α = 0.89, respectively).

#### Statistical Analyses

Double data entry was performed to ensure data reliability. All statistical analyses were performed using the software Statistical Package for the Social Sciences (SPSS) version 22.

Prior to analyses, missing data (<1%) in questionnaires were replaced with the participant’s mean score on the respective measure. All measures were then screened for univariate and multivariate normality, and distributions of all continuous data were checked. Distributions on measures were considered normal according to figures of skew and kurtosis. Generally, scores were normally distributed with all items demonstrating acceptable levels of skewness and kurtosis (≤|1| ). The DASS-21 Anxiety and Depression scales evidenced significant skewness and were subsequently transformed to a normal distribution by applying a log10 transformation. Internal consistency of all measures was assessed by computing Cronbach alphas (α) coefficients.

Intercorrelations among all study measures were performed by calculating Pearson’s *r* or point-biserial coefficients. Mediation models were then tested using a bootstrapping approach through the PROCESS macro for SPSS (Hayes, 2012, 2013). PROCESS, in addition to testing traditional path coefficients, provides direct, indirect and total effects, and bias corrected and accelerated confidence intervals (CI). Mediation generally occurs when 95% CI of the indirect effect estimated from the bootstrap procedure excludes zero. Hayes (2013) demonstrated that bootstrapping allows for more accurate and powerful analyses than traditional mediation approaches (e.g., Baron and Kenny, 1986); furthermore, differently from traditional mediation approaches, modern approaches such as bootstrapping do not require statistically significant direct effects to interpret indirect effects (Hayes, 2013). Two mediation analyses were performed. In each model, IU (IUS-12) was entered as the independent variable; worry (PSWQ) and somatic anxiety (DASS-21 Anxiety) were, in turn, entered as dependent variables; three parallel mediators, i.e., PBW (WW-III), NPO (NPOQ), and CA (R-CAQ), were entered simultaneously. Notably depression, as measured by the DASS-21 Depression scale, was entered as a covariate in each model in light of the strength of the associations emerged with all the models’ variables (please refer to **Table 2**). For each analysis, we used 5,000 bootstrap samples; 99% bias corrected CIs to evaluate the significance of the conditional indirect effects were used in light of the numerous comparisons being done in multiple samples.

Furthermore, since PROCESS allows testing conditional indirect effects of an independent variable, which means assessing an independent variable’s effect on the dependent variable through mediators, depending on a moderator (i.e., moderated mediation) (Hayes, 2012), we conducted *post hoc* analyses including IU (IUS-12) as the moderator when appropriate.

### Results

#### Descriptive Statistics and Correlational Analyses

Mean scores (SDs) obtained by participants (not transformed scores) on all the study’s measures are reported in **Table 1**. Correlations between all measures are reported in **Table 2**. As shown, there were positive correlations between all measures (small–medium range). Furthermore, the relations of age and gender to all variables were assessed. Age was not correlated with any of the study measures (all *p*s > 0.05), whereas gender (coding: 0 = male; 1 = female) was weakly related, in terms of effect size, to the PSWQ (*r* = 0.20, *p* = 0.009).

**Table 1:** Mean, standard deviations and Cronbach alphas observed in the UK undergraduate sample in all study measures.

	*M*	*SD*	Cronbach alpha
IUS-12	30.38	10.47	0.93
WW-III	91.36	22.97	0.94
NPOQ	30.99	12.50	0.96
R-CAQ	85.77	3.66	0.94
PSWQ	52.49	14.94	0.78
DASS-21 Anxiety	3.89	4.04	0.85
DASS-21 Depression	4.66	4.25	0.89

*IUS-12, Intolerance of Uncertainty Scale-12; WW-III, Why Worry-III; NPOQ, Negative Problem Orientation Questionnaire; R-CAQ, Revised-Cognitive Avoidance Questionnaire; PSWQ, Penn State Worry Questionnaire; DASS-21 Anxiety, Depression Anxiety Stress Scale–Anxiety subscale; DASS-21 Depression, Depression Anxiety Stress Scale–Depression subscale.*

**Table 2:** Correlations (Pearson rs) between the scores on all measures observed in the UK undergraduate sample.

	WW-III	NPOQ	R-CAQ	PSWQ	DASS-21 Anxiety	DASS-21 Depression
IUS-12	0.19**	0.70**	0.47**	0.68**	0.53**	0.50**
WW-III		0.20**	0.26**	0.28**	0.19**	0.23**
NPOQ			0.49**	0.68**	0.53**	0.58**
R-CAQ				0.36**	0.40**	0.40**
PSWQ					0.49**	0.48**
DASS-21 Anxiety						0.59**

IUS-12, Intolerance of Uncertainty Scale-12; WW-III, Why Worry-III; NPOQ, Negative Problem orientation Questionnaire; PSWQ, Penn State Worry Questionnaire; DASS-21 Anxiety, Depression Anxiety Stress Scale–Anxiety subscale; DASS-21 Depression, Depression Anxiety Stress Scale–Depression subscale. **p < 0.001.

#### Mediation Models

**FIGURE 1:**
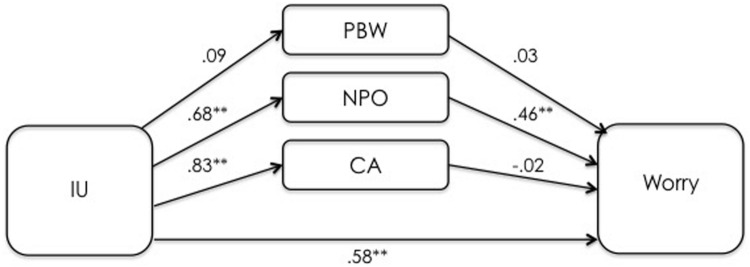
**Unstandardized regression coefficients between IU, mediators, and worry in the UK undergraduate sample.** **p < 0.001.

The first mediation analysis examined the indirect effects of IUS-12 scores on PSWQ scores through WW-III, NPOQ, and R-CAQ, controlling for DASS-21 Depression. Unstandardized regression coefficients of the direct effects are reported in **Figure 1**.

**FIGURE 2:**
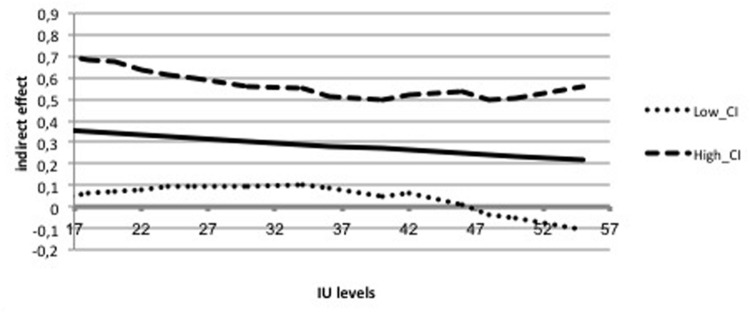
**Conditional indirect effect of IU on worry through NPO in the UK undergraduate sample**.

**FIGURE 3:**
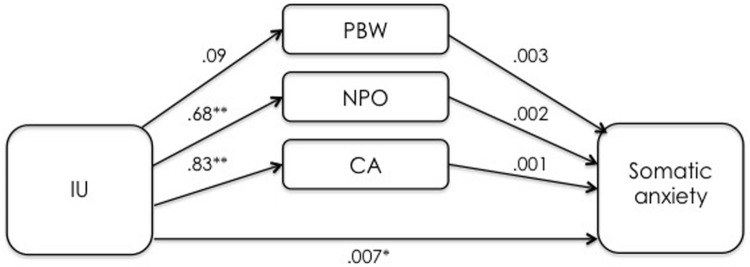
**Unstandardized regression coefficients between IU, mediators, and somatic anxiety in the UK undergraduate sample.** **p < 0.001.

The overall model was significant (*F*_5,164_ = 45.44, *p* < 0.001) and explained 58.1% of the variance in PSWQ scores. The total effect of IUS-12 scores on PSWQ scores was significant (*b* = 0.8836, *SE* = 0.0887, *t* = 9.97, *p* < 0.001, 99% CIs = 0.6526, 1.1146). The covariate DASS-21 Depression was not significant (*p* = 0.42). Specific and total indirect effects are shown in **Table 3**. The indirect effect of IUS-12 on PSWQ through NPOQ was significant. *Post hoc* moderated mediation analyses were then performed. The IUS-12 × NPOQ interaction, i.e., the conditional indirect effect (**Figure 2**) was not significant (*b* = -0.0053, *SE* = 0.0065, *t* = -0.81, *p* = 0.42, 99% CIs = -0.0223, 0.0117). The second mediation analysis examined the indirect effects of IUS-12 scores on DASS-21 Anxiety scores through WW-III, NPOQ, and R-CAQ, controlling for DASS-21 Depression. Unstandardized regression coefficients of the direct effects are reported in **Figure 3**. The overall model was significant (*F*_5,164_ = 25.26, *p* < 0.001), and explained 43.5% of the variance in DASS-21 Anxiety scores. The total effect of IUS-12 scores on DASS-21 Anxiety was significant (*b* = 0.0104, *SE* = 0.0023, *t* = 4.57, *p* < 0.001, 99% CIs = 0.0045, 0.0164), and the covariate DASS-21 Depression resulted significant (*p* < 0.001). Specific and total indirect effects are shown in **Table 3**. None of the indirect effects was significant. In light of results, *post hoc* moderated mediation analyses were not conducted.

**Table 3:** Specific and total indirect effects of IU on worry and somatic anxiety through PBW, NPO, and CA controlling depression in the UK undergraduate sample.

				Bootstrapped 99% CIs	
Dependent variable	Mediators	Point estimate	*SE*	Lower	Upper
PSWQ	WW-III	0.0029	0.0112	-0.0196	0.0626
	NPOQ	0.3152	0.0810	0.1220	0.5729
	R-CAQ	-0.0208	0.0339	-0.1252	0.0702
	Total	0.2972	0.0810	0.1138	0.5287
DASS-21-Anxiety	WW-III	0.0000	0.0002	-0.0005	0.0011
	NPOQ	0.0019	0.0018	-0.0027	0.0068
	R-CAQ	0.0010	0.0010	-0.0016	0.0041
	Total	0.0029	0.0021	-0.0020	0.0089

*WW-III, Why Worry-III; NPOQ, Negative Problem Orientation Questionnaire; R-CAQ, Revised-Cognitive Avoidance Questionnaire; PSWQ, Penn State Worry Questionnaire; DASS-21 Anxiety, Depression Anxiety Stress Scale–Anxiety subscale.*

### Discussion

Results from the first mediation model suggest that only NPO has a mediational role in the path from IU and worry in UK undergraduates. This finding provides only partial support to the original hypotheses, since neither PBW nor CA emerged to mediate the relationship between IU and worry. Furthermore, results from the second mediation model highlighted that the path from IU to somatic anxiety was not mediated by any of the three hypothesized mediators. Despite not confirming our hypotheses, such a result could be reasonable considering that the Laval model of GAD aimed to conceptualized PBW, NPO, and CA as specifically related to worry severity than to somatic anxiety symptom severity. Lastly, IU did not emerge to influence the mediational path to worry through NPO, thus it was not a moderator.

## Study 2

### Materials and Methods

#### Participants and Procedure

Four hundred and eighty-eight Italian undergraduate students (61.7% females), aged between 19 and 27 years (*M* = 21.17, *SD* = 1.54), voluntarily entered the study. All of them were Caucasian. Participants were all attending their 2nd year of university studies at the Schools of Psychology and Engineering of the University of Padova (Italy). They all provided their written informed consent before completing the study measures; no incentives were offered for participation. Participants filled in a socio-demographic schedule and then questionnaires were administered in counterbalanced order to control for order effects. The research was approved by the Ethics Committee of the Psychological Sciences, University of Padova.

#### Measures

The Italian translations of the above-described (please refer to Study 1) IUS-12, WW-III, NPOQ, and R-CAQ were administered. Since no validated version of those instruments was available at the time the study was conducted, the standard forward-back translation procedure was performed (e.g., Brislin, 1986). Three independent researchers translated the questionnaires from English to Italian and then reached agreement on a common version for each measure. Idiomatic Italian at the 6th-grade level was used for this step. Moreover, the researchers reviewed the common version to ensure there were no colloquialisms, slang, or esoteric phrases that would make interpretations difficult. The shared forms were then back-translated by a bilingual individual with extensive knowledge of psychological research. The back-translations proved to be nearly identical to the original ones. As a final step, 5 experts in anxiety disorders rated the items of all the newly developed Italian versions. Each expert rated the items on a 5-point scale (1 = not at all, 5 = extremely) for clarity (the extent to which the item is clearly described). The experts’ ratings indicated excellent clarity, indicating that further item refinement was unnecessary. Notably, preliminary evidence of good psychometric properties of the Italian translations of these measures was provided (Bottesi et al., 2014). Moreover, a preliminary validation of the Italian IUS-12 on a sample of university students showed that it possesses good internal consistency, construct and discriminant validities, and gender invariance (Bottesi et al., 2015b). In the present study, all the measures showed acceptable (α > 0.60; Theorell et al., 1993) to excellent (α > 0.90) internal consistency values (please see **Table 4**).

**Table 4:** Mean, standard deviations and Cronbach alphas observed in the Italian undergraduate sample in all study measures.

	*M*	*SD*	Cronbach’s alpha
IUS-12	24.63	6.44	0.81
WW-III	93.39	25.21	0.94
NPOQ	24.50	8.93	0.92
R-CAQ	71.61	18.78	0.93
PSWQ	43.83	11.27	0.68
BAI	9.15	8.48	0.90
BDI-II	9.65	8.23	0.90

*IUS-12, Intolerance of Uncertainty Scale-12; WW-III, Why Worry-III; NPOQ, Negative Problem Orientation Questionnaire; R-CAQ, Revised-Cognitive Avoidance Questionnaire; PSWQ, Penn State Worry Questionnaire; BAI, Beck Anxiety Inventory; BDI-II, Beck Depression Inventory–Second Edition.*

Furthermore, the validated Italian version of the PSWQ (Morani et al., 1999) was administered. In the current sample, internal consistency was acceptable (α = 0.68).

Lastly, instead of the DASS-21, the Beck Anxiety Inventory (Beck et al., 1988; Italian version by Sica et al., 2006) and the Beck Depression Inventory-II (Beck et al., 1996; Italian version by Ghisi et al., 2006) were employed to measure somatic anxiety and depressive symptoms, respectively.

The *Beck Anxiety Inventory* (BAI; Beck et al., 1988; Italian version by Sica et al., 2006) is a 21-item self-report questionnaire measuring the severity of anxiety over the previous week. Participants are required to answer on a 4 point-Likert scale ranging from 0 = “not at all” to 3 = “severely-I could barely stand it).” The BAI possesses excellent internal consistency and good 1-week test–retest reliability (Beck et al., 1988). The Italian version of the BAI demonstrated good internal consistency and 30-day test–retest reliability (Sica et al., 2006; Sica and Ghisi, 2007); internal consistency was excellent in the study sample (α = 0.90).

The *Beck Depression Inventory-II* (BDI-II; Beck et al., 1996; Italian version by Ghisi et al., 2006) is a 21-item self-report questionnaire assessing the severity of affective, cognitive, motivational, vegetative, and psychomotor components of depression. Each BDI-II item is rated on a 4-point scale ranging from 0 to 3 and the instructions ask the respondent to endorse, for each item, the most characteristic statement, covering the time frame of “the past 2 weeks, including today.” The BDI-II showed high internal consistency and good 1-week test–retest reliability among college students (Beck et al., 1996). The Italian version evidenced excellent psychometric properties as well (Sica and Ghisi, 2007), and the Cronbach alpha value observed in the present sample was α = 0.90.

#### Statistical Analyses

The same procedure and data analytic strategy described in Study 1 were applied in Study 2. Also in Study 2 several variables, namely NPOQ, R-CAQ, BAI, and BDI-II evidenced significant skewness and were subsequently transformed to a normal distribution by applying a log10 transformation.

Furthermore, similar to Study 1, the employed depression measure (BDI-II) showed positive correlations with all the other variables (please refer to **Table 5**) and was therefore included as a covariate into the two mediation models.

**Table 5:** Correlations (Pearson rs) between the scores on all measures observed in the Italian undergraduate sample.

	WW-III	NPOQ	R-CAQ	PSWQ	BAI	BDI-II
IUS-12	0.34**	0.57**	0.35**	0.51**	0.40**	0.41**
WW-III		0.25**	0.29**	0.38**	0.23**	0.23**
NPOQ			0.43**	0.56**	0.46**	0.58**
R-CAQ				0.35**	0.43**	0.42**
PSWQ					0.53**	0.49**
BAI						0.57**

IUS-12, Intolerance of Uncertainty Scale-12; WW-III, Why Worry-III; NPOQ, Negative Problem orientation Questionnaire; PSWQ, Penn State Worry Questionnaire; BAI, Beck Anxiety Inventory; BDI-II, Beck Depression Inventory–Second Edition. **p < 0.001.

### Results

#### Descriptive Statistics and Correlational Analyses

Means (SDs) and Cronbach alphas obtained on all the study measures (not transformed scores) are displayed in **Table 4**.

**Table 5** shows correlations between questionnaires within the Italian sample. Results highlighted positive correlations between all measures (small–medium range). Furthermore, age emerged to be weakly negatively related only with the BAI (*r* = -0.14, *p* = 0.002), and gender was weakly related, in terms of effect size, to the PSWQ (*r* = 0.16, *p* < 0.001) and the BAI (*r* = 0.18, *p* < 0.001). Age and gender were not associated with scores obtained in the other study measures (all *p*s > 0.05).

#### Mediation Models

**FIGURE 4:**
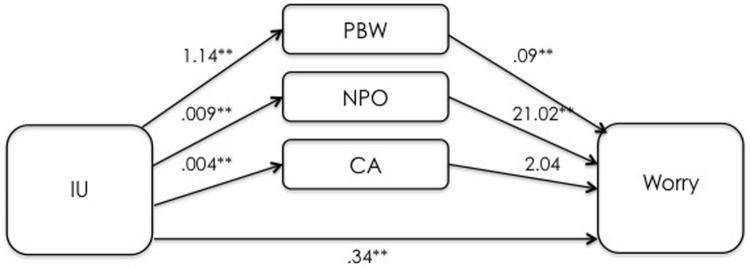
**Unstandardized regression coefficients between IU, mediators, and worry in the Italian undergraduate sample.** **p < 0.001.

**FIGURE 5:**
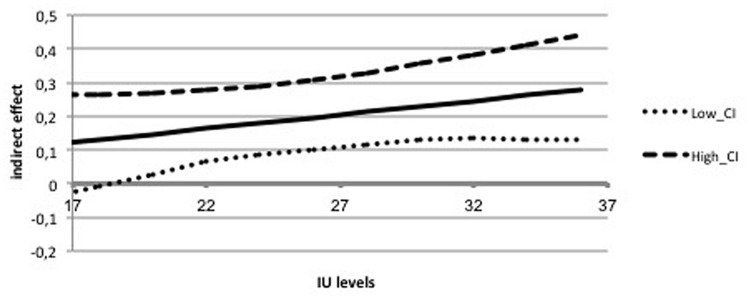
**Conditional indirect effect of IU on worry through NPO in the Italian undergraduate sample**.

**FIGURE 6:**
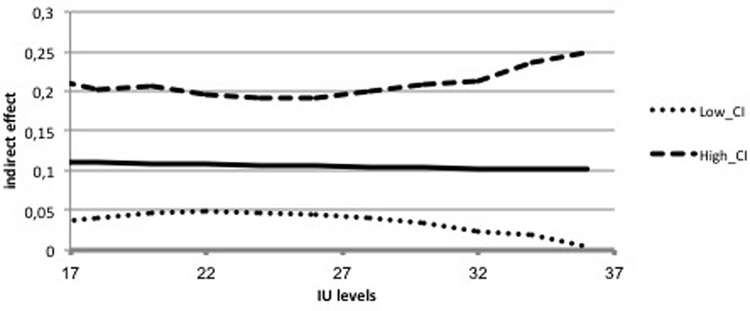
**Conditional indirect effect of IU on worry through PBW in the Italian undergraduate sample**.

The first mediation analysis examined the indirect effects of IUS-12 scores on PSWQ scores through WW-III, NPOQ, and R-CAQ scores, controlling for BDI-II scores. Unstandardized regression coefficients of the direct effects are reported in **Figure 4**. The overall model was significant (*F*_5,482_ = 74.88, *p* < 0.001) and explained 43.7% of the variance in PSWQ scores. The total effect of IUS-12 scores on PSWQ scores was significant (*b* = 0.64, *SE* = 0.07, *t* = 9.20, *p* < 0.001, 99% CIs = 0.4596, 0.8190). The covariate BDI-II also resulted significant (*p* < 0.001). Specific and total indirect effects are shown in **Table 6**. The indirect effects of IUS-12 to PSWQ through WW-III and NPOQ were significant. *Post hoc* moderated mediation analyses were then performed. Findings highlighted that the IUS-12 × NPOQ interaction, i.e., the conditional indirect effect, was slightly significant and positive (*b* = 0.90, *SE* = 0.47, *t* = 1.92, *p* = 0.049, 99% CIs = 0.3107, 2.1192), thus suggesting that the path is moderated by IUS-12 scores. A visual inspection of results emerged from the bootstrapped tests (**Figure 5**) shows that the indirect effect of NPOQ scores was increasing with IUS-12 scores over almost all of IUS-12 scores range. On the contrary, the IUS-12 × WW-III interaction (**Figure 6**) emerged not to be significant (*b* = -0.0005, *SE* = 0.0026, *t* = -0.18, *p* = 0.86, 99% CIs = -0.0072, 0.0062).

**Table 6:** Specific and total indirect effects of IU on worry and somatic anxiety through PBW, NPO, and CA controlling depression in the Italian undergraduate sample.

				Bootstrapped 99% CIs	
Dependent variable	Mediators	Point estimate	*SE*	Lower	Upper
PSWQ	WW-III	0.0989	0.0462	0.1888	0.4313
	NPOQ	0.1909	0.0377	0.0990	0.2958
	R-CAQ	0.0077	0.0157	-0.0298	0.0555
	Total	0.2975	0.0462	0.1888	0.4313
BAI	WW-III	0.0005	0.0007	-0.0013	0.0024
	NPOQ	0.0019	0.0012	-0.0011	0.0051
	R-CAQ	0.0022	0.0007	0.0007	0.0045
	Total	0.0046	0.0015	0.0009	0.0086

*WW-III, Why Worry-III; NPOQ, Negative Problem Orientation Questionnaire; R-CAQ, Revised-Cognitive Avoidance Questionnaire; PSWQ, Penn State Worry Questionnaire; BAI, Beck Anxiety Inventory.*

**FIGURE 7:**
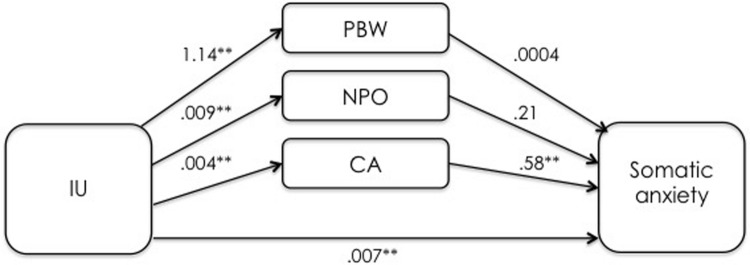
**Unstandardized regression coefficients between IU, mediators, and somatic anxiety in the Italian undergraduate sample.** **p < 0.001.

**FIGURE 8:**
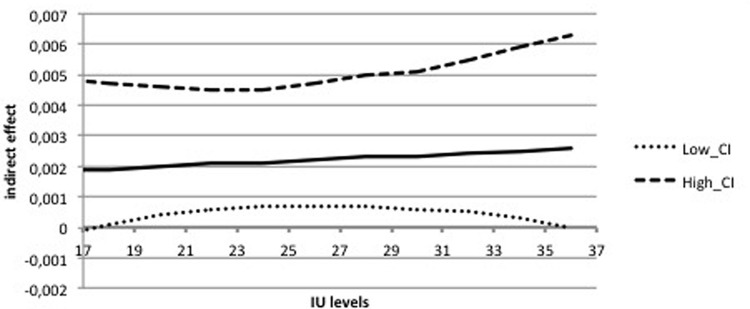
**Conditional indirect effect of IU on somatic anxiety through CA in the Italian undergraduate sample**.

The second mediation analysis examined the indirect effects of IUS-12 scores on BAI through WW-III, NPOQ, and R-CAQ scores controlling for BDI-II scores. Unstandardized regression coefficients of the direct effects are reported in **Figure 7**. The overall model was significant (*F*_5,482_ = 61.93, *p* < 0.001) and explained 39.1% of the variance in BAI. The total effect of IUS-12 scores on BAI scores was significant (*b* = 0.0119, *SE* = 0.0023, *t* = 5.14, *p* < 0.001, 99% CIs = 0.0059, 0.0179). The covariate BDI-II resulted significant (*p* < 0.001). Specific and total indirect effects are shown in **Table 6**. The indirect path of IUS-12 on BAI through R-CAQ was significant. Also in this case, *post hoc* moderated mediation analyses were conducted. The IUS-12 × R-CAQ interaction, i.e., the conditional indirect effect (**Figure 8**), was not significant (*b* = 0.0090, *SE* = 0.0215, *t* = 0.42, *p* = 0.68, 99% CIs = -0.0466, 0.0646).

### Discussion

Findings emerged from the two mediation models were partially in line with our hypotheses. First, both PBW and NPO, but not CA, were significant mediators in the path from IU and worry; second, only CA (but not NPO) played a mediational role in the path from IU and somatic anxiety. Lastly, IU moderated only the association between NPO and worry: The higher were IU levels, the larger was the mediational effect of NPO.

## General Discussion

The IUM for GAD identified four main components associated with the development and maintenance of worry: IU, PBW, NPO, and CA (Dugas et al., 1998). Among them, IU was conceptualized as a background factor and the literature of the last 20 years supports the notion of IU as a vulnerability factor for worry (Dugas et al., 1998; Ladouceur et al., 2000; Sexton et al., 2003; Koerner and Dugas, 2008). Despite studies showing the contributions of PBW, NPO, and CA and occasionally in a mediational path from IU to worry (e.g., Dugas et al., 2007; Koerner and Dugas, 2008), whether and how these components can turn IU into worry and somatic anxiety still remains to be clarified. Therefore, the current studies aimed at gathering a better understanding of the original Laval model by testing two mediation models using bootstrapping approaches in two university samples made up of UK and Italian undergraduate students.

Our first hypothesis stated that PBW would have mediated the relationship between IU and worry but not the relationship between IU and somatic anxiety. As far as concern the path to worry, such an expectation was only partially sustained: Indeed, PBW emerged as a mediator only in the Italian sample. The different mediational patterns observed across samples is no better explained by a different degree of reliance in such beliefs across samples, since groups scored similarly on the WW-III (*F*_1,657_ = 0.85, *p* = 0.36). Nonetheless, it is noteworthy that PBW have been proposed to initially drive the worry process, thus fostering the initial use of worry as a coping strategy and, once established across time, their role is variable (Dugas et al., 2007). Consequently, in light of the higher levels of both IU (*F*_1,657_ = 70.61; *p* < 0.001; $\eta_{p}^{2}$ = 0.10) and worry (*F*_1,657_ = 62.20, *p* =< 0.001; $\eta_{p}^{2}$ = 0.09) demonstrated by the UK students when compared with the Italian ones, one could hypothesize that a greater establishment of worry – altogether with a minor endorsement of PBW – might have already occurred in the former and, therefore, PBW could have already played their function. As regards the path from IU to somatic anxiety, consistently with our hypotheses, PBW emerged not to play a mediational role in both cultures; this confirms that, given their content specificity, PBW are logically associated with worry (Freeston et al., 1994).

The second hypothesis of the current study was that NPO and CA would have been mediators in the paths from IU to both worry and somatic anxiety. As far as concern NPO, it emerged to play a mediational role in the path from IU to worry both in Studies 1 and 2 thus demonstrating its involvement across cultures. On the other hand, NPO did not emerge to play any mediational role in the path from IU to somatic anxiety, which is in contrast with previous evidence highlighting this cognitive variable to be relevant also to somatic anxiety (e.g., Dugas et al., 2007; Fergus and Wu, 2011). Nonetheless, as outlined in other studies, NPO (as well as PBW and CA) are theorized to contribute directly to worry, which is then posited to contribute to somatic anxiety symptoms (e.g., Dugas and Robichaud, 2007; Dugas et al., 2007). In light of this, our result is barely surprising. An opposite pattern of results emerged when analyzing the role of CA. Indeed, findings revealed that it mediated only the path from IU to somatic anxiety and only in the Italian sample. Its involvement in the association between IU and somatic anxiety provides further support to the evidence stating that CA strategies are relevant in affecting peripheral activation and somatic symptoms (e.g., Borkovec et al., 2004), even though understanding why this emerged only in the Italian student sample is struggling. Notably, also in this case it is to note that UK undergraduates showed higher levels of CA than Italian ones (*F*_1,657_ = 62.29; *p* < 0.001; $\eta_{p}^{2}$ = 0.09), therefore hypothesizing such a finding as a function of a differential endorsement of CA strategies across samples is not explanatory. Furthermore, since the two samples were administered different measures of anxiety symptoms (i.e., the DASS-21 Anxiety in the UK sample and the BAI in the Italian one), speculating about differences between samples in relation to somatic anxiety levels is not possible. As already mentioned, CA emerged not to mediate the path from IU to worry in both samples. This is quite an unexpected finding, considering that CA is considered an important maintenance factor of worry (Borkovec et al., 1993, 2004; Dugas et al., 1998), and raises questions about the involvement of this component into the model.

*Post hoc* moderated mediation analyses were performed, when appropriate, to test whether IU levels moderated the mediational effect of the other components in the paths to worry and somatic anxiety. Findings revealed that IU emerged as a moderator only in the path to worry through NPO, and only in Italian students, thus providing only partial support to the idea of IU as a moderator of this relationship. Such a difference could be cautionary explained in terms of cross-cultural issues. Indeed, whilst UK has been reported as a culture whose members are more prone to take and manage unknown risks, Italy is included among the “strong uncertainty avoidance cultures” (De Mooij, 2000, 2003; Stremersch and Tellis, 2004; Wennekers et al., 2007). Notably, economic and financial crises have troubled Italy in the last decade; this might have somewhat impacted on a population (and on its young adult portion in particular) supposed to be resistant to change and particularly conservative. Notably, this does not necessarily mean that Italian university students are more intolerant of uncertainty than their UK counterparts; rather, they are likely to attribute a different (and more negative) meaning to uncertainty and/or to be less used to it (as demonstrated by scores obtained on the IUS-12, where scores shown by the Italian undergraduates were even significantly lower than those obtained by UK undergraduates). Nonetheless we acknowledge that, overall, our findings failed to demonstrate that the contribution of PBW, NPO, and CA depends on the level of IU, thus not providing additional help in understanding why higher IU leads to higher levels of worry (e.g., Ladouceur et al., 2000; Koerner and Dugas, 2008).

To summarize, the current re-examination of the IUM for GAD showed that the original conceptualization of the model, claiming that IU contributes to worry and anxiety through PBW, NPO, and CA is substantially supported but only in the Italian undergraduate sample, whereas findings emerged from the UK one are controversial. The discrepancies characterizing main results obtained in the two samples further strengthen the recommendation of performing cross-cultural studies in the field of anxiety, since it looks like that people from different cultures might actually interpret and express their symptoms in a different way, and cross-cultural research should help in understanding why and how (Malgady, 1996; Kirmayer, 2001; Norton, 2005). For example, related to this and with specific regard to IU, Norton (2005) explored the psychometric properties and the structure factor of the IUS in four different racial groups (African, Caucasian, Hispanic, and Southeast Asian) and findings showed that, despite substantially similar reliability and validity values across the four racial groups emerged, differences in the factor structures across groups were found; the extent to which cultural or racial variables had influenced results was not clearly definable but could not be excluded. A number of issues might have contributed in determining different patterns across samples and should be acknowledged as weaknesses characterizing the present research. First, the UK undergraduate sample was mainly made up of female participants (86.5%), whereas the Italian one was only slightly unbalanced in regard to gender (61.7% females). Such a disproportion in terms of gender composition may have affected results, since evidence highlighting that women are more likely to refer fear and higher levels of physiological hyperarousal, catastrophic cognitions, and anxiety sensitivity than men have been reported in literature (e.g., Armstrong and Khawaja, 2002; McLean and Anderson, 2011). On the other hand, in the present study female gender emerged to be (weakly) associated with higher scores on the PSWQ in both samples (and with scores obtained on the BAI in the Italian sample), therefore the two samples appear to be similarly characterized despite the number of females included. Possibly related to this, UK students showed higher levels of IU, NPO, CA, and worry than their Italian counterparts. Such differences could be reasonably attributed to a gender effect (but interactions Group × Gender for all measures resulted *p* > 0.05); to a “subject effect,” since the Italian sample was made up not only of Psychology students like the UK sample, but also of Engineering students who can show both a different approach and sensitivity to “psychology stuff”; to a culturally different attitude to metacognition; to different mechanisms intervening in different cultures. Indeed, emerged findings could also suggest that UK students are more prone to worry than Italian ones, but the processes through which they worry are different. Future studies should further investigate these topics. Another already mentioned difference between samples is in terms of measures employed to assess somatic anxiety and depression levels; this limits the possibility of tracing reliable comparisons between the results emerged in the two samples. Nonetheless, excellent convergent validity values between the DASS-21, the BAI, and the BDI-II have been extensively reported across cultures (Antony et al., 1998; Daza et al., 2002; Bados et al., 2005; Bottesi et al., 2015a), thus suggesting that such instruments substantially measure the same constructs. Furthermore, it is to note that scores ranges observed in the two samples on these measures were in line with previous literature on large non-clinical samples (e.g., Henry and Crawford, 2005; Sica and Ghisi, 2007).

Several further limitations characterizing the present study should be taken into account. The sample sizes employed, despite not extremely small, do not allow to generalize emerged results. A further obstacle to the generalization of results is represented by the cross-sectional nature of the current data. Indeed, we tested a conceptually (and partially empirically demonstrated) plausible model, but many cross-sectional models have equally plausible alternatives; furthermore, a number of biases characterizing the tests of mediation when using cross-sectional data have been highlighted (e.g., Maxwell and Cole, 2007). Therefore, longitudinal research in this field is highly recommended. Moreover, given the nature of samples, studies testing the same models also in community as well as in clinical populations are advocated. Indeed, whilst the adequacy and the advantages of employing analog samples when conducting research on clinical phenomena has been claimed (e.g., Ruscio et al., 2001; Olatunji et al., 2010; Abramowitz et al., 2014), current findings are not necessarily generalizable to other populations and, therefore, need to be intended as a starting point for additional research focusing on differently characterized samples. Another shortcoming is represented by the fact that some of the measures administered in the UK sample were not-validated refinements of previous versions (i.e., the WW-III and the R-CAQ), and some questionnaires used in the Italian sample were translations – and not proper validations – of the correspondent English ones (e.g., the IUS-12, the WW-III, the NPOQ, and the R-CAQ); nevertheless, internal consistency values were adequate both in the present study and in previous researches (Bottesi et al., 2014, 2015b). An additional issue deserving attention is the decision we made about partialling out depression in the models we tested. The rationale for doing so was eliminating a third variable scenario (depression) and increasing the specificity of findings to worry and somatic anxiety; however, in the current study measures of depression correlated with measures of worry and somatic anxiety in the 0.48 to 0.59 range and partialling out depression certainly took out a lot of variance. Therefore, such an issue raises questions about what is the portion of worry/anxiety that does not overlap with depression conceptually: Future developments should consider controlling for anhedonia rather than for shared features. Related to this, it is worthy to further highlight that increasing evidence supporting the notion of IU as a transdiagnostic factor involved in several psychopathologies beyond GAD, such as obsessive compulsive disorder (e.g., Tolin et al., 2003; Holaway et al., 2006), depression (e.g., Buhr and Dugas, 2002; Dugas et al., 2004; van der Heiden et al., 2010), post-traumatic stress disorder (e.g., Banducci et al., 2016; Oglesby et al., 2016), panic disorder and agoraphobia (e.g., McEvoy and Mahoney, 2012; Carleton et al., 2013), and social anxiety (e.g., Riskind et al., 2007; Boelen and Reijntjes, 2009; Carleton et al., 2010), has been documented. Future studies aiming to achieve a more in-depth knowledge of the commonalities that may exist between disorders are recommended; moreover, investigating the functioning of high-level factors, such as IU, in different phenomenologies might increase our understanding of comorbidities (Gentes and Ruscio, 2011).

Keeping in mind the above-mentioned limits, overall present results provide support to the current refinement of the original Laval model, where the IUM components are better conceptually integrated. Differential cross-cultural IU mechanisms, as well as potentially different worry domains across cultures, might have contributed to observed findings and deserve further investigation. Clinically, the findings tentatively suggest that understanding how people of different cultures understand and express uncertainty and IU may be central in our ability to target it effectively in therapy.

## Author Contributions

GB: Performed literature review, statistical analyses and wrote the manuscript. MG: Performed literature review and contributed in writing up the manuscript. EC, NB, and RP: Performed data collection, data entry, and support to data analyses. MF: Designed and supervised the entire project.

## Conflict of Interest Statement

MF declares he has received training honoraria and book royalties on closely related topics. All the other authors declare that the research was conducted in the absence of any commercial or financial relationships that could be construed as a potential conflict of interest.
